# Genome sequence of *Alteromonas macleodii* strain OCN004 isolated from the extracellular mucus of an apparently healthy rice coral (*Montipora capitata*)

**DOI:** 10.1128/mra.00079-24

**Published:** 2024-02-23

**Authors:** Elizabeth F. Weatherup, Patrick Videau, Blake Ushijima

**Affiliations:** 1 Department of Biology and Marine Biology, University of North Carolina Wilmington, Wilmington, North Carolina, USA; 2 Department of Biology, Southern Oregon University, Ashland, Oregon, USA; Montana State University, Bozeman, Montana, USA

**Keywords:** coral, *Alteromonas*, genome

## Abstract

*Alteromonas macleodii* strain OCN004, a marine gammaproteobacterium in the *Alteromonadaceae* family, has primarily been studied as a non-pathogenic negative control bacterium during laboratory infection trials to test the virulence of bacterial coral pathogens. The draft genome sequence of *A. macleodii* strain OCN004 is presented here.

## ANNOUNCEMENT

*Alteromonas macleodii* is a Gram-negative, aerobic marine gammaproteobacterium within the *Alteromonadaceae* family found in a wide range of marine environments (1–3). *Alteromonas* strains have been studied for their enzymatic activities (4), natural product production (5), and probiotic treatments for shellfish pathogens (6, 7); however, no *A. macleodii* strain has been described as a pathogen to date. *A. macleodii* strain OCN004 (hereafter OCN004) has been used as a negative control in fulfilling the Henle-Koch postulates to demonstrate that three *Vibrio* and a *Pseudoalteromonas* strains are etiological agents of coral disease (8–10), and in the study of virulence genes and chemotaxis in coral pathogens (11, 12). As coral disease poses a significant threat to reefs worldwide, sequencing non-infectious bacterial control strains used in coral research is a critical component of improving the understanding of disease dynamics and members of the coral holobiont.

The extracellular mucus of an apparently healthy *Montipora capitata* coral from the reef around Moku o Loʻe in Kāneʻohe Bay, Hawaiʻi, USA (21.432659 N, −157.787358 W) was diluted in sterile filtered seawater, spread on glycerol artificial seawater (GASW) medium solidified with 1.5% agar, and OCN004 was picked from and purified on this medium as previously described (8). OCN004 was routinely cultured on GASW buffered with 50 mM Tris base (pH 8.3) and incubated overnight at 27°C (12). The isolation of genomic DNA was conducted using a plate culture derived from a single colony by the SeqCenter, LLC (Pittsburgh, PA, USA) using the ZymoBIOMICS DNA Miniprep Kit according to the manufacturer’s instructions (Irvine, CA, USA). Libraries were prepared using the tagmentation- and PCR-based Illumina DNA Prep kit (Illumina, San Diego, CA, USA) and custom IDT 10 bp unique dual indices (UDI) with a target insert size of 320 bp. Sequencing resulted in 2,795,576 151 bp paired-end reads from an Illumina NovaSeq 6000 sequencer. FastQC (http://www.bioinformatics.babraham.ac.uk/projects/fastqc) was used to assess read quality; trimming and adapter sequence removal were performed using BBDuk within the BBMap package (http://sourceforge.net/projects/bbmap) as previously described (13). The draft genome sequence was assembled with SPAdes v. 3.15.0 using the “--isolate” option (14). The genome was analyzed for completeness using BUSCO v. 5.2.2 with default parameters and the bacteria_odb10 and alteromonadales_odb10 databases (15). Average nucleotide identity (ANI) and *in silico* DNA-DNA hybridization (isDDH) determinations were conducted using JSpecies and GGDC (16–18), respectively, with 95%–96% ANI and 70% isDDH used as the minimum values to define novel species (17, 19–23).

The assembly produced 93 scaffolds with a mean coverage of 39.1× and an N_50_ value of 136,227 bp. The draft genome sequence comprises a total of 4,463,050 bp with a 44.6% G + C content. BUSCO scores for the genome were 99.2% and 99.9% for the bacterial (123/124 genes) and *Alteromonadales* (819/820 genes) gene sets, respectively. Genomic analyses revealed that OCN004 shares 97.0% ANI and 78.0% isDDH with *A. macleodii* strain ATCC 27126^T^ (12). These genomic indices of relatedness are above the cutoff values for species delineation, which indicates that OCN004 is a strain of *A. macleodii*.

## Data Availability

This whole-genome shotgun project was deposited in DDBJ/ENA/GenBank under the accession number JAWQJS000000000. The version described in this paper is version JAWQJS000000000.1. Raw sequence reads were deposited in the SRA under accession number SRS19498708 and are associated with BioSample number SAMN38027836.
